# Total knee Arthroplasty: Patient expectations and outcomes in a resource constrained setting

**DOI:** 10.12669/pjms.42.3.13328

**Published:** 2026-03

**Authors:** Muhammad Ahmed Ghazni Khan, Mohammad Ahsan Sulaiman, Mohammad Motiwala, Shahryar Noordin

**Affiliations:** 1 Muhammad Ahmed Ghazni Khan Senior Registrar Orthopedics, Memon Medical Institute Hospital, Hyder Buksh, Safoora Chowrangi, Gabol Road, Karachi, Pakistan; 2 Mohammad Ahsan Sulaiman Department of Orthopedics, Aga Khan University, Stadium Road, Karachi 74800, Pakistan; 3 Mohammed Motiwala Dean Clinical Research Fellow, Department of Surgery, Aga Khan University, Stadium Road, Karachi 74800, Pakistan; 4 Shahryar Noordin Department of Orthopedics, Aga Khan University, Stadium Road, Karachi 74800, Pakistan

**Keywords:** Total knee arthroplasty, Functional outcome, Patient-Reported Outcome Measures (PROMs), Patient expectations vs outcomes

## Abstract

**Objective:**

Knee osteoarthritis significantly impacts the quality of life, particularly in advanced stages. Total knee arthroplasty (TKA) is a common treatment, but 20-30% of patients remain dissatisfied despite clinical improvements. This study evaluates TKA outcomes using patient-reported outcome measures (PROMs) in a low-middle-income country context.

**Methodology:**

A prospective cohort study from Aga Khan University Hospital of 70 patients with end-stage knee osteoarthritis undergoing TKA was conducted from May, 2023 to December, 2023, Improvement in Knee injury and Osteoarthritis Outcome Score (KOOS-42) at three months post-TKA was assessed. The secondary objectives were to evaluate the correlation between patient expectations and outcomes, a comparison of unilateral and bilateral TKA, and the effects of patellar resurfacing and home-based physiotherapy. Data were analyzed using Stata 17.0.

**Results:**

Significant improvements in KOOS scores were observed three months post-TKA (mean 71.0 ± 7.3, P<0.001) compared to preoperative scores (26.3 [21.6-30]). Patient expectations were higher than actual postoperative outcomes (P<0.001). The pre-operative functional score and Patellar resurfacing improved symptom scores (P=0.003), while other factors like unilateral vs. bilateral TKA and home-based physiotherapy showed no significant impact on overall KOOS scores.

**Conclusion:**

TKA significantly improves patient-reported outcomes, but a gap remains between expectations and actual outcomes. Tailored patient education is crucial to manage expectations and enhance satisfaction, especially in resource-limited settings. These findings support the need for patient-centered care approaches in TKA.

***Abbreviation:***

**TKA:** Total Knee Arthroplasty. **KOOS:** Knee injury and Osteoarthritis Outcome Score. **PROMs:** Patient-Reported Outcome Measures. **KOOS PES**: Knee Injury and osteoarthritis (Patient expectations score). **LMICs:** Low- and Middle-Income Countries. **BMI:** Body Mass Index. **ERC:** Ethical Review Committee. **GEE:** Generalized Estimating Equation. **QOL:** Quality of Life. **OA:** Osteoarthritis. **HB:** Hemoglobin. **HCT:** Hematocrit.

## INTRODUCTION

Knee osteoarthritis, a globally prevalent condition affecting an estimated 22.9% of individuals over 40,^1^ significantly diminishes the quality of life in advanced stages, causing persistent pain, reduced mobility, and potential complications from long-term analgesic use.^1^-^3^Total knee arthroplasty (TKA) is a common and effective treatment, but despite clinical improvement, 20-40% of patients remain dissatisfied, highlighting a gap in understanding and managing patient expectations.^4^-^7^ This is particularly critical in low-middle-income countries, where high patient volumes and limited resources often hinder comprehensive patient care.^8^ The assessment of total knee arthroplasty (TKA) outcomes has primarily relied on surgeon-determined measures, such as knee range of motion, implant stability, and postoperative alignment. However, studies have revealed a discrepancy between surgeon-derived outcomes and those reported by patients, with surgeons tending to overestimate outcomes compared to patients’ assessments.^9^ This suggests a need to incorporate patient-reported outcome measures (PROMs) more prominently. Since Total knee arthroplasty is an elective surgery aimed at improving a patient’s quality of life, patient-reported outcome is hugely important for how well TKA has helped patients achieve their desired goals.

### Primary Objective:

- To evaluate improvement in the KOOS-42 score at three-month intervals after total knee arthroplasty (TKA), as the greatest improvement typically occurs within this period.

### Secondary Objectives:

- To assess the correlation between patient expectations and actual outcomes at three months post-TKA.
- To compare postoperative KOOS scores between unilateral and bilateral TKA.
- To determine the effect of patellar resurfacing versus non-resurfacing on postoperative KOOS scores.
- To evaluate the impact of home-based supervised physiotherapy on patient-reported outcomes following TKA.

The three-months follow-up was chosen to capture significant early postoperative improvements in patient-reported outcome measures (PROMs) and to facilitate timely interventions for suboptimal outcomes, thereby optimizing patient recovery.^10^

### Focus on LMICs:

Understanding the impact of patellar resurfacing and supervised physiotherapy in low- and middle-income countries (LMICs) like Pakistan is critically important. In these settings, access to specialized healthcare services and postoperative supervised rehabilitation is often limited due to the scarcity of dedicated rehabilitation centers. Additionally, patellar resurfacing increases the overall implant cost, which further adds to the financial burden on patients. Therefore, evaluating the necessity of such interventions in LMICs is essential to ensure accessible, sustainable, and patient-centered orthopedic care.

## METHODOLOGY

This was a single-centered prospective cohort study from Aga Khan University Hospital, on patients who underwent total knee replacement at a tertiary care center from May 2023 to October 2023 and was commenced after approval from the institutional ethical review committee (ERC 2023-8346; dated: May 30, 2023).

### Inclusion criteria:

- All patients above 40 years old, diagnosed with end-stage OA on clinical and radiological parameters, and undergoing total knee arthroplasty with consent to take part in this study were included.

### Exclusion criteria:

- Patients who either declined to consent or those who underwent revision arthroplasty, and complex knee arthroplasty with constrained implants were excluded from this study. Additionally, individual who became lost to follow-up were also excluded.

### Sampling technique:

Nonprobability consecutive sampling technique was employed.

For sample size estimation we used the formula:


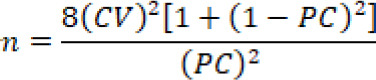


**n:** The required sample size, **CV:** Coefficient of variation, **PC:** Pearson correlation coefficient

The minimum sample size required was 70 patients who underwent total knee arthroplasty with an inflation of 30 % for loss to follow-up/non-response rate, to achieve 80% power with at least 20% change in mean Knee injury and osteoarthritis outcome and 40% or less change in the coefficient of variation at two-sided 5% level of significance.

### Data collection tool:

Patient-reported outcome measures (PROMs) are widely used to assess surgical outcomes, offering unbiased patient perspectives and insight into modifiable factors^8^, ^9^ . This study used the validated Urdu version of KOOS, proven reliable and responsive for evaluating functional disability in knee osteoarthritis patients.

To assess patient expectations before surgery, patients completed the KOOS questionnaire indicating their anticipated score at three months post-TKA, termed the KOOS Patient Expectation Score (KOOS-PES). Comparing KOOS-PES with postoperative KOOS outcomes provided valuable insight into patients’ expectations and their alignment with actual results, offering a more patient-centered measure of surgical success.

### Data Collection Procedure:

After obtaining informed consent, demographic and clinical data including age, gender, body mass index (BMI), comorbidities, and laterality of surgery were recorded by an orthopedic resident. Preoperatively, patients completed the KOOS-42 questionnaire, with assistance provided as needed, and the KOOS Patient Expectation Score (KOOS-PES) at admission to document expected outcomes. Postoperatively, the KOOS-42 was re-administered at the three months follow-up. Patients unable to complete the forms due to illiteracy or visual impairment were assisted by trained staff without influencing responses. All patients received daily physiotherapy from the first postoperative day, including knee range-of-motion, ankle pump, and quadriceps-strengthening exercises. At discharge, patients chose between assisted home physiotherapy by a trained therapist or self-directed physiotherapy as taught during hospitalization. The KOOS-42 is a joint-specific patient-reported outcome measure in which patients are instructed to answer based on the operated knee. In unilateral TKA, patients responded with reference to the operated knee only, while in bilateral TKA, responses reflected the overall function of both operated knees. To minimize the influence of the contralateral knee in unilateral cases, trained research staff provided clear verbal instructions during questionnaire administration, limiting assistance to clarification without influencing responses.

### Statistical analysis:

Stata version 17.0 was used for data entry, management, and analysis. The following binary variables “home physiotherapy regimen, patellar resurfacing, and tourniquet removal” were calculated using the individual protocols of each surgeon. Preliminary checks included a Shapiro-Wilk test for continuous variables to assess data distribution for continuous variables. Summary statistics and one-way association tables were then generated for continuous and categorical variables, respectively.

For continuous variables, paired t-tests and Wilcoxon rank-sum tests were employed for normally distributed and non-parametric data, respectively. Given the prospective nature of the study, a generalized estimating equation (GEE) was utilized for comprehensive analysis.

## RESULTS

Total 70 patients diagnosed with end-stage knee osteoarthritis participated in this study, comprising predominantly females (73%). Their average age was 61.4 years, with an average weight of 76.9 kg and a mean BMI of 31.4kg/m^2^ Table-I. Among these participants, 61% underwent unilateral total knee replacement, 77% had patellar resurfacing, 66% had tourniquet removal before wound closure, and 19% were prescribed a post-operative home physiotherapy regimen.

**Table-I:** Overall patient characteristics among patellar resurfacing and non-resurfacing groups.

Characteristics	Overall	Patellar Resurfacing		p-Value
		NO N (%) n=16	YES N (%) n=54	
Age	61.4 ± 8.9	61.75 ± 7.6	61.2 ± 9.4	0.849
Sex				
Female	51 (73%)	12 (75%)	39 (72%)	0.826
BMI	31.4 ± 5.0	33.5 ± 4.3	30.8 ± 5.0	0.062
Height (cm)	155 [149-161]	155.5 [149-160]	155 [149-162]	1.000
Weight (kg)	76.9 ± 13.2	79.8 ± 10.2	76.0 ± 14.0	0.316
Duration of tourniquet (minutes)	80 [70-132]	78 [72.5-143]	80.5 [69-127]	0.586
Side operated (Unilateral)	43 (61%)	9 (56%)	34 (63%)	0.628
Tourniquet removal (Before)	46 (66%)	12 (75%)	34 (63%)	0.373
KOOS PES (expectation)	89 [85-91.6]	90.7 [87.5-92.7]	87.5 [83.3-90]	0.078
KOOS 42 admission	26.3 [21.6-30]	27.4 [23.3-36.6]	25.5 [21.6-29]	0.168

BMI: Body Mass Index, KOOS PES: Knee Injury and osteoarthritis (Patient expectations score).

### Preoperative And Postoperative Assessments:

**Fig.1:**
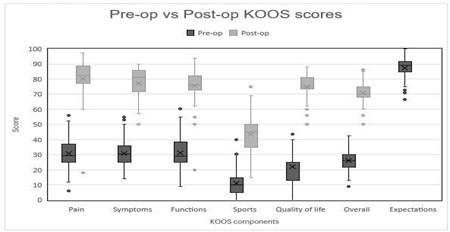
Comparison of preoperative and postoperative KOOS 42 scores by component.

**Fig.2:**
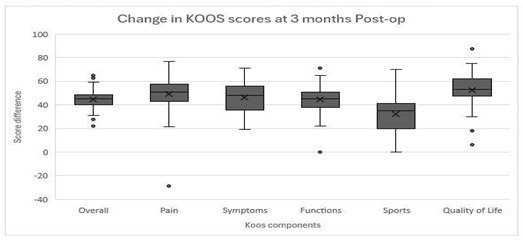
Change in KOOS 42 scores from preoperative and postoperative by component.

Preoperative assessment using the KOOS 42 questionnaire revealed notably low scores with a median score of 26.3 [21.6-30]. The quality of life (QOL) component at admission was particularly low, averaging 22.2 ± 10. Conversely, the KOOS-Patient expectations score exhibited significantly higher scores, indicating optimistic expectations with a median score of 89 (p<0.001). Following surgery, at the Three-months follow-up period, there was a substantial improvement in KOOS scores, reaching a mean of 71.0 ± 7.3 (P<0.001) Fig.1 and 2. It was also noted that there was a consistent trend (96%) of expectation scores being generally higher than the actual three-month KOOS scores (P<0.001).

### Factors affecting outcomes:

Various surgical parameters-such as timing of tourniquet removal, unilateral versus bilateral procedures, home-based postoperative physiotherapy, and patellar resurfacing-did not significantly influence the overall KOOS component scores, except for the Symptom component of KOOS-42 at three months in the grouping based on patellar resurfacing (P=0.003) Table-II.

**Table-II:** Postoperative KOOS 42 scores at 3 months grouped by patellar resurfacing.

Outcomes	Overall	Patellar Resurfacing		p-Value
		NO N (%) n=16	YES N (%) n=54	
Overall KOOS score	71.0 ± 7.3	68.5 ± 6.8	71.7 ± 7.3	0.130
Pain	82.5 [77.7-88.9]	78 [70.5-85]	83 [78-88.9]	0.092
Symptoms	81 [72-85.7]	70.5 [63-77.5]	81 [75-86]	0.003
Functions	76 [73-82.4]	77.5 [73.5-81.9]	76 [72-82.4]	0.865
Sports	43.6 ± 11.9	42.5 ± 9.7	43.9 ± 12.5	0.688
Quality of Life	75 [75-81.3]	75 [66.5-78.1]	75 [75-81.3]	0.530

### Generalized estimating equation analysis:

### Regression Analysis:

Backward stepwise multivariable linear regression was done on Post-Op KOOS 42 with patient characteristics, pre-op KOOS 42 components, and expectation scores. The pre-op function score coefficients of (non-normal distribution) 0.313 [95% CI 0.142-0.484] and patellar resurfacing coefficient of 4.764 [95% CI 1.042-8.487] were significantly associated with a higher post-op score, p-values 0.001 and 0.013 respectively. A trend of increasing post-op score was seen with the KOOS expectation score with a coefficient of 0.209 [95% CI -0.042-0.460] which was also a non-normally distributed P-value (0.101) Table-III.

**Table-III:** Generalized Estimating Equation for predictors of Koos outcome score.

Variables	Adjusted Coefficient [95% CI]	P-value
Age	0.021 [-0.124 - 0.167]	0.772
Female	0.011[-3.174 - 3.197]	0.995
Unilateral TKA	-4.200 [-10.474 - 2.074]	0.190
Tourniquet opening after closure	-1.722 [-6.976 - 3.533]	0.521

### Key Findings:

The study highlights a marked improvement in KOOS scores three months post-total knee replacement surgery compared to preoperative scores. It also underscores the disparity between patient expectations, which tended to be higher, and the actual postoperative outcomes measured by KOOS. Patellar resurfacing and preoperative function KOOS assessments significantly impacted perceived postoperative outcomes.

## DISCUSSION

As the number of TKR performed is progressively increasing^8^,^11^ and focus of care has progressively moved towards patient-centered care, there has been a significant need to understand the expectations and hence cater effectively to patient needs. Our study, which comprises a series of patients who underwent total knee arthroplasty (TKA) for end-stage osteoarthritis attempts to understand at a quantitative level, the discrepancy between expectations and outcomes in the patient population at our center.

The patient characteristics in our study are comparable with other studies assessing TKA outcomes in terms of age, gender, and BMI^4^,^12^,^13^ and no significant differences between patients within groups of patellar resurfacing, tourniquet removal timing, and unilateral versus bilateral surgery; forming one of this study’s key strengths.

The median admission KOOS 42 falling within the lower limit of the poor classification (Table-II), reflects the extent of impairment in the lives of TKA patients emphasizing the necessity of intervention. The mean KOOS 42 at three months postoperatively, averaging 71.0 ± 7.3 falls within the fair classification. The improvement in KOOS 42 overall score compared to the preoperative KOOS aligns with findings from other studies assessing the impact of TKA.^14^ The most notable average difference in KOOS scores between preoperative vs postoperative individual components was observed in quality-of-life scores, with a score of 53 [CI 47.5-62], followed by pain 51 [CI 43-57.8] and symptoms 48 [35.7-56]. This comprehensive improvement across all aspects of the KOOS score indicates a holistic improvement in the patient’s condition after surgery and serves as a robust indicator of the success of TKA surgeries.^13^,^15^,^16^

The study emphasizes a notable disparity between preoperative expectations which were significantly higher than both the preoperative KOOS 42 26.3 [21.6-30] and the subsequent postoperative KOOS 42 scores, a difference of 17 [11.9-21.6]. This consistent trend has been observed in prior research utilizing different assessment tools, where expectations tend to surpass the actual outcome scores.^14^,^17^,^18^ Common expectations in those studies include pain relief, improved walking, engagement in sports, recreational activities, and daily activities of living. Scott CE et al found that expectations related to kneeling, squatting, and stair climbing, ranked 4^th^ 9^th,^ and 13^th^ respectively in importance by patients, were not met despite an 87% satisfaction rate among a sample of 323 patients undergoing TKA.^19^ Therefore, while TKA significantly enhances quality of life, it falls short of fulfilling all expectations from surgery.

This also highlights the need to understand factors influencing patient satisfaction and dissatisfaction. Kahlenberg et al in a systematic review have shown that higher functional scores most commonly predict satisfaction followed by reduced pain and greater improvement in function scores from preoperative to postoperative assessments.^20^ Conner-Spady et al revealed that a significant proportion of unmet expectations pertained to physical activities and sports, postulating a potential discordance in expectations between surgeons and patients.^21^ However, it is worth noting that the cited studies predominantly originate from developed countries like Canada, highlighting the potential benefits of conducting qualitative studies on expectations in operations within low-middle-income countries (LMICs) to improve concordance and patient care.

### Subgroup analysis:

The results show no significant impact of unilateral vs simultaneous bilateral knee replacement on postoperative KOOS scores. This aligns with a comprehensive systematic review of 19 studies with 191,094 patients, revealing negligible differences in complication rates and functional scores.^22^,^23^ This finding can guide surgeons to choose the most appropriate techniques for their patients, whether unilateral or bilateral, without worrying about a significant effect on postoperative functional outcomes.

The issue of patellar resurfacing has sparked debates, with recent literature suggesting limited utility in certain cases such as patellofemoral osteoarthritis, inflammatory arthropathy, and patellar malalignment. Routine resurfacing is discouraged due to prolonged operation times and increased risk of complications.^24^ In our study, the impact of patellar resurfacing was confined to the symptom component of KOOS 42 with average scores of 70.5 versus 81 (p=0.003) in the non-resurfacing and the resurfacing groups respectively; however, no significant difference carried over to the overall score with patellar resurfacing alone.

### Strengths:

Prospective design, validated Urdu KOOS tool, and assessment of both patients reported outcome and patient expectations.

### Limitations:

Small sample size, single-center data, and short-term (three-month) follow-up. Future multicenter studies with longer follow-up are recommended to assess sustained improvement and satisfaction trends. Short follow-up period, designed to understand outcomes in the early post-op period when significant improvements in PROM are typically observed. It facilitates timely intervention if any suboptimal outcome arises, enabling healthcare providers to address issues promptly and optimize patient outcomes.

## CONCLUSION

This study from a low and middle-income country underscores the importance of patient expectations in total knee arthroplasty outcomes. Significant improvement in KOOS scores at three months demonstrated substantial gains in pain, function, and quality of life. Patellar resurfacing and home-based supervised physiotherapy did not significantly affect overall KOOS outcomes, suggesting comparable recovery without added costs. These findings support a more sustainable, patient-centered approach to knee arthroplasty in resource-constrained settings

### Author’s contributions:

**MAGK, AS and MM:** Data collection, and analysis.

**AGK, AS and MM:** Prepared the first draft of the manuscript was written.

**SN:** Supervision of the study.

**MAGK:** Responsible for the accuracy of the study.

All authors commented on previous versions of the manuscript read and approved the final manuscript.

All authors contributed to the study’s conception and design. Manuscript preparation.
